# Model of Environmental Membrane Field for Transmembrane Proteins

**DOI:** 10.3390/ijms22073619

**Published:** 2021-03-31

**Authors:** Irena Roterman, Katarzyna Stapor, Piotr Fabian, Leszek Konieczny, Mateusz Banach

**Affiliations:** 1 Department of Bioinformatics and Telemedicine, Jagiellonian University—Medical College, 30-688 Kraków, Poland; mateusz.banach@uj.edu.pl; 2 Institute of Computer Science, Silesian University of Technology, 44-100 Gliwice, Poland; katarzyna.stapor@polsl.pl (K.S.); piotr.fabian@polsl.pl (P.F.); 3 Chair of Medical Biochemistry, Jagiellonian University—Medical College, 31-034 Kraków, Poland; mbkoniec@cyf-kr.edu.pl

**Keywords:** hydrophobicity, mechanosensitive channel, membrane, ion channel, water/membrane environment

## Abstract

The water environment determines the activity of biological processes. The role of such an environment interpreted in the form of an external field expressed by the 3D Gaussian distribution in the fuzzy oil drop model directs the folding process towards the generation of a centrally located hydrophobic core with the simultaneous exposure of polar residues on the surface. In addition to proteins soluble in the water environment, there is a significant group of membrane proteins that act as receptors or channels, including ion channels in particular. The change of the polar (water) environment into a highly hydrophobic (membrane) environment is quite radical, resulting in a different hydrophobicity distribution within the membrane protein. Modification of the notation of the force field expressing the presence of the hydrophobic environment has been proposed in this work. A modified fuzzy oil drop model with its adaptation to membrane proteins was used to interpret the structure of membrane proteins–mechanosensitive channel. The modified model was also used to describe the so-called negative cases—i.e., for water-soluble proteins with a clear distribution consistent with the fuzzy oil drop model.

## 1. Introduction

The level of protein solubility depends on the exposure of polar residues to the protein surface. The concept of a hydrophobic core is associated with the central concentration of hydrophobic residues with the simultaneous exposure of polar residues on the surface [1]. Even a single mutation—especially changing the local level of hydrophobicity/polarity—can change the solubility and even completely change the structural form of the protein. Here, the classic example is sickle hemoglobin, where a single E6V mutation in the Beta chain completely changes the quaternary structure of hemoglobin [2,3]. Another example of protein structural differentiation is transthyretin, a protein that undergoes amyloid transformation. Two forms of this protein have been distinguished: aggressively undergoing the amyloid transformation process [4] and the form resistant to this process [5]. This differentiation proves a different tendency to aggregate these two forms in the presence of two and three mutations in relation to the wild-type form of this protein. 

Apart from the mutation influencing the folding of the polypeptide chain, the characteristics of the environment in which the process takes place have a decisive influence on the folding process and the final hydrophobicity distribution. The water environment promotes micelle-like structuring (centric concentration of hydrophobicity with simultaneous exposure of polar residues on the surface). An environment with opposite properties for proteins is the cell membrane representing a system with a high level of hydrophobicity. The influence of the environment on the structuring of proteins can be observed in the example of membrane proteins, including proteins that act as channels for the transport of molecules. 

Membrane anchored proteins pose considerable difficulties in structural studies due to the requirements of traditional X-ray-based methods related to the solubility condition [6]. Therefore, the synthesis of membrane proteins in a cell-free environment turns out to be a way to recognize their structures [7,8]. In this field, methods of the numerical prediction of membrane protein structures also have their achievements [9,10,11,12,13]. 

The cell membrane, apart from the sheath function ensuring cell cohesion, is the site of activity of numerous proteins, especially receptors [6,14]. The water environment and the hydrophobic environment in the membrane are a combination of two separate external force fields, preferring the opposing order of the residues from the point of view of their polarity. Due to this differentiation, proteins anchored in cell membranes are an interesting object of analysis [15]. The main group of membrane proteins are proteins that act as transport channels for various components, in particular ions. [16]. Besides the exposure of hydrophobicity on the surface (contact with membrane), the concentration of polar residues is observed in the central part of molecule—the channel is located in the central part [17]. The purpose of the analysis presented here is to assess the diversity of hydrophobicity distributions in proteins consisting of two parts: anchored in the membrane and exposed to the cytoplasmic space. 

The analysis of the hydrophobicity distribution also allows for speculation about the protein-folding process itself, which, taking its native structure, adapts to the properties and characteristics of the environment [18].

The use of the fuzzy oil drop model [19] enables the quantification of the degree of adjustment to the idealized distribution in the form of 3D Gaussian distribution reveals the diversity of the specificity of proteins. The complexation of the second protein is favored by the exposure of the hydrophobic surface [20] and the possibility of ligand complexation, often related to function or interaction with the substrate, as is the case with enzymes [21]. It is associated with local hydrophobicity deficiency. The given examples of inconsistencies of the idealized distribution (consistent with the 3D Gaussian distribution) are often identified as local. Elimination of single residues showing significant differences between the theoretical and the observed distribution results in obtaining the status of the rest of the molecule consistent with the distribution showing the presence of a centric hydrophobic core with a polar shell. 

The analysis of amyloid structures based on the fuzzy oil drop model showed the specificity of the structural forms of these proteins in the form of matching the hydrophobicity distribution to the 2D Gaussian form due to the flat structure of individual chains present in amyloid fibrils [22].

In the present work, a modification of the fuzzy oil drop model was proposed to enable identification and comparative analysis of membrane protein structures. The introduced modification was tested on the example of membrane proteins from the group of the mechanosensitive channel—MscS [23,24,25,26,27]. A “negative” analysis was also performed applying the correction for water-soluble proteins [28,29]. This is to verify the universality of the amendment introduced. The modification of the field did not change the assessment of the status of water-soluble proteins and proteins showing the local incompatibility of the distribution observed with the idealized distribution.

## 2. Theory—Modification of the Fuzzy Oil Drop Model—Introduction of the Hydrophobic Environment 

The fuzzy oil drop model assumes the distribution of the hydrophobicity of a water-soluble globular protein as a specific micelle-like structure. The structure of micelles made of bi-polar molecules arises spontaneously by exposing the polar parts to the environment with the simultaneous concentration of parts of hydrophobic molecules in the central part of the micelles. As a result of such a process, the hydrophobicity density distribution can be described by means of the 3D Gaussian distribution, where the maximum of this function located in the center of the sphere expresses the highest concentration of hydrophobicity.

To answer the questions “why the authors developed this ‘fuzzy oil drop’ model”, “what it can do that could not be done before”, and “what novel insights their model has”, two aspects should be taken under consideration.

First, in computer simulation, the presence of an environment for protein folding is realized in the form of a certain number of individual water molecules, which interact with folding proteins in the form of atom–atom interactions. The fuzzy oil drop model represents the external force field in a continuous form directing the hydrophobic residues towards the central part of the protein body with simultaneous exposure of polar residues on the surface. This is why it represents the synergy in the process of folding, making the water environment an active partner in the folding process.

Second, the final effect of folding may represent the micelle-like status with regular highly ordered hydrophobicity distribution. However, not all proteins are able to reach the status of such a high order. The degree of accordance/discordance can be measured quantitatively and the localization of the discordance region can be identified. Thus, the proteins can be classified according to the degree of reaching micelle-like construction. This is how they may be compared revealing their specificity. On the other hand, the simulation of proteins in the external force field may visualize the active participation of water in the folding process. The participation of the water environment can be modified in a heuristic manner expressing changes in the characteristics of its surroundings for the folding process, as is demonstrated in this paper. 

The polypeptide chain consists of 20 types of amino acids characterized by differentiated levels of hydrophobicity. This chain in an aqueous environment, undergoing a process similar to the micellarization process, generates a structure characterized by the exposure of polar amino acids on the surface, and the concentration of hydrophobic residues in the central part of the molecule. The degree of matching of the distribution by the polypeptide chain to that obtained by micelles consisting of free-moving molecules is possible only to a limited extent due to the deterministic character of amino acids sequence and covalent bonds between them, making free movement impossible. The fuzzy oil drop model comparing the idealized hydrophobicity distribution, consistent with the 3D Gaussian distribution present in a given protein, and the distribution resulting from hydrophobic interactions between amino acids, enables a quantitative assessment of the order of the micelle-like hydrophobicity distribution.

The idealized distribution—T—is determined by defining the appropriate 3D Gaussian distribution (parameters sigmaX, sigmaY, and sigmaZ are adjusted to the size of the molecule). The value of the 3D Gaussian function in the so-called effective atom (average position of atoms making up a given amino acid) determines the level of hydrophobicity in the position of a given amino acid.

The level actually present in a position of a given effective atom position is determined by the Levitt function [30]. The value of the observed hydrophobicity level (Oi) depends on the distance between the interacting amino acids and their intrinsic hydrophobicity. Both these distributions after their normalization (the sum of all *T_i_* equal to 1 and the sum of all *O_i_* equal to 1) can be compared using the Kullback–Leibler entropy divergence—*D_KL_* [31] expressed as:

(1) $$D_{KL}=\sum_{i=1}^{N}O_{i}\log_{2}\left(O_{i}/T_{i}\right)$$

The *D_KL_* (also expressed as *O|T* later in this paper to make the form shorter) value also expresses the distance between the distribution *T* and *O*. The distribution *T* plays the role of the reference distribution. The value determined in this way, however, cannot be interpreted—it expresses entropy. Therefore, a second reference distribution—*R*—is introduced, where each residue (each effective atom) has a hydrophobicity level assigned equal to 1/N where N is the number of amino acids in the chain. Such a distribution expresses a state in which no concentration of hydrophobicity in the form of a hydrophobic core is present. A *D_KL_* value for the *O|T* relation lower than the *D_KL_* value for the *O|R* relation indicates the presence of a hydrophobic core in the molecule. In order to not use two values for the description of the same object, the parameter RD (relative distance) was introduced, expressed as follows:

(2) $$RD_{T-O-R}=\frac{O/T}{O/T+O/R}$$

An *RD* value less than 0.5 indicates the presence of a hydrophobic core. The notation *O|T* means the value of *D_KL_* for the relation *O* in relation to the distribution *T* as a reference, *O|R* means the value of *D_KL_* for the relation *O* with reference distribution *R*. The value of *RD* can be determined for structural units of the type: complex, single-chain, or domain. In each of these cases, a 3D Gaussian function is generated for each of the units listed.

It is also possible to evaluate the status of a selected chain fragment as chain components. The new 3D Gaussian function is then not generated. The *T* and *O* and *R* profiles for a selected segment within a given form of the 3D Gaussian function subjected to normalization make it possible to evaluate the status of the selected fragment.

A protein with *RD* > 0.5 is interpreted as having no hydrophobic core. Gradually eliminating the residues showing the greatest difference between *T_i_* and *O_i_* leads to a status of *RD* < 0.5. In this way, it is possible to assess the causes of discrepancies. It turns out that the residues showing a local excess of hydrophobicity often interact with another chain, eliminating unfavorable contact of the hydrophobic surface with the polar environment, being part of the interface [20]. The local hydrophobicity deficit is often associated with the presence of a cavity—the ligand or substrate-binding site [21]. 

It should be emphasized that the expected presence of the micelle-like hydrophobicity distribution is the effect of the environment, especially the water environment. Polar water molecules direct the folding process towards the centralization of hydrophobic residues. The simulation of the folding process with the active participation of an external force field in the form of a 3D Gaussian function leads to structuralization creating the presence of a hydrophobic core [32]. Using the 3D Gauss function to represent the environment makes the external force field continuous, in contrast to other models representing the water environment as a set of individual water molecules. 

The fuzzy oil drop model has already been described [33]. Its repeated description in the present work results from introducing a modification concerning the influence of a non-aquatic environment on the generation of protein structures. We are talking about the environment of the cell membrane. The force field, which is the cell membrane, can be defined as the opposite of the water environment. The presence of a hydrophobic environment implies a different organization process of bipolar amino acids. In a hydrophobic environment, it is the hydrophobic residues that are expected on the surface of the protein. Thus, the polar residues should rather be directed towards the interior of the molecule.

**Figure 1:**
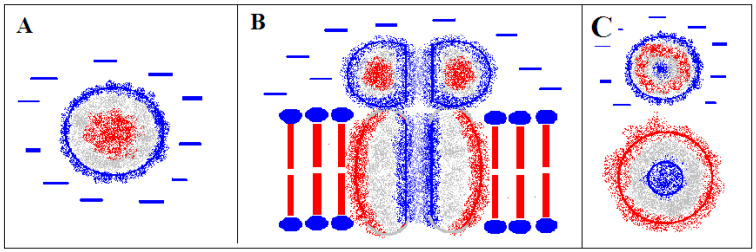
Model of hydrophobicity distribution in. (**A**)—globular protein with a hydrophobic core structure of micelle-like type operating in a water environment (**B**)—membrane protein, that is, operating in a hydrophobic environment with a centrally located ion channel present (lower domain), the presence of a channel with a polar coat in the domain directed towards the cytoplasm. The red bars represent the hydrophobic fragments of the membrane components, the blue circles—the polar groups of molecules that make up the membrane. (**C**)—cross-sections of the two domains of the membrane protein shown in B. Colors used—red—hydrophobicity, blue—polarity, gray—intermediate zone.

Proteins belonging to the membrane channel group are abundantly represented membrane proteins. These channels are used to transport various molecules, including ions in particular. The channel located in the central part of the molecule anchored in the membrane additionally suggests the presence of polar groups on the channel walls. Schematically, the situation in the globular protein and in the membrane protein serving as a channel (including ion channels in particular) is shown in Figure 1.

The analysis of the hydrophobicity distribution symbolically shown in Figure 1A suggests the use of a 3D Gaussian function to describe the distribution in globular proteins operating in an aqueous environment. The concentration of hydrophobicity present in the central part of the molecule with the surface covered with a polar shell creates favorable conditions for the solubility of the protein. It is also the effect of the active participation of the external field in the process of folding the polypeptide chain, directing this process towards the preferred exposure of polar residues on the surface and the concentration of hydrophobic residues in the center. This type of hydrophobicity distribution is represented by the 3D Gaussian function Figure 1B,C). 

**Figure 2:**
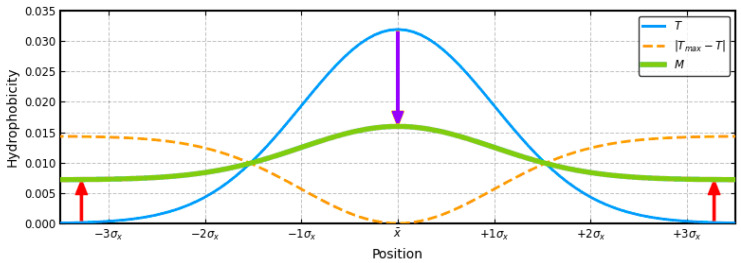
The functions applied in the FOD-M model in step-wise form. Blue line—globular proteins with the hydrophobicity of the form of micelle-like expressed by the 3D Gauss function. Concentration of hydrophobicity in the central part with the polar residues on the surface—hydrophobicity approach zero level—*T* distribution. Orange dashed line—idealized hydrophobicity distribution in membrane-channel protein—the distribution “inversed” with the lowest hydrophobicity in the central part (channel) with high hydrophobicity on the surface (contact with membrane)—(*T_ma_*_x_ − *T_i_*) *_n_*. Green line—consensus between 3D Gauss function and “inversed” function expressing the synergy between polar and hydrophobic environment. Red arrows—increase of hydrophobicity on the surface. Blue arrow—decrease of the hydrophobicity level in the central part.

On the other hand, the distribution of hydrophobicity in the membrane protein acting as an ion channel suggests a distribution “opposite” to that of 3D Gauss. On the surface, high hydrophobicity (compatibility with the membrane environment) and polarity in the center where the channel is located are expected. The “inverse” distribution was written as Max-3DG where Max is the maximum value of Ti hydrophobicity in a given 3D Gauss distribution. This is explained in Figure 2. 

The blue line in Figure 2 shows a Gaussian distribution favoring the formation of a centric hydrophobic core (reduced to one dimension). The orange dashed line (Figure 2) expresses the expectation of high hydrophobicity on the surface, while the lowest level of hydrophobicity is expected in the central part of the molecule (of course if we take the cross-section perpendicular to the long axis of the molecule—the ion channel). In the membrane-anchored part, the mismatch in the distribution within the protein (domain) concerns the central part, where the polar channel contradicts the expectation of the presence of a hydrophobic core. 

However, domains exposed to the water environment are covered with a polar surface. The mismatch here is the presence of a polar center at the site of the expected high hydrophobicity concentration. 

The final form of the external field notation (Figure 2—green line) takes into account the influence of the hydrophobic environment on a significant increase in the level of hydrophobicity in the zones close to the surface and a significant decrease in the level of hydrophobicity in the central part, where the channel is located (free space with a polar surface) and is written in the form:

M_i_ = [T_i_ + (T_max_ − T_i_)_n_]_n_ (3)

where *Mi*—the level of hydrophobicity resulting from the presence of the membrane environment assigned to the *i*-th residue. *T_max_* denotes the maximum value for the *T* distribution (distribution in form of 3D Gauss function). Index n denotes the normalization of the distribution. 

Taking the Mi distribution as the reference distribution for O the *D_KL_* value can be calculated.

(4) $$D_{KL}=\sum_{i=1}^{N}O_{i}\log_{2}\left(O_{i}/M_{i}\right)$$

In consequence, the *RD* for T-O-M relation is as follows:

(5) $$RD_{T-O-M}=\frac{O/T}{O/T+O/M}$$

**Figure 3:**
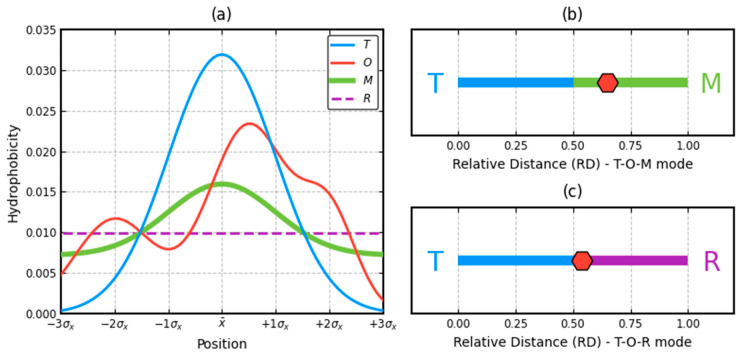
Analysis of examples (reduced to one-dimensional simplified version) of distribution (**a**) *O* (red) in respect to theoretical (*T*—blue) and membrane influence distribution (M—green) and the reference distribution *R* (violet dashed). Values of *RD* (relative distance) for (T-O-M) = 0.651 (**b**) and for (T-O-R) = 0.539 (**c**).

The effect of the modification is also shown in Figure 3. The *O* distribution compared with *T* and *M* distribution (reduced for simplicity to one dimension presentation) expressed in *RD* values is shown in Figure 3b,c.

Summarizing the modification of the fuzzy oil drop model, which also extends the application of this model to proteins with a significantly different structure compared to micelle-like, it should be stated that the [(*T_MAX_* − *T_i_*)*_n_*] component expresses the presence of a factor changing the standard characteristic for the polar environment, i.e., the impact of changes in environmental conditions on the construction of a molecule with a different structural form in relation to the molecule subjected only to the external field, which is the polar aquatic environment. The modification introduces a component expressing the hydrophobic surrounding. 

## 3. Results 

Analysis of discussed proteins is focused on the interpretation of RD parameters for T-O-R and T-O-M relations. A low value of RD suggests a structure with a hydrophobic core in the central part of the molecule (domain, chain, complex). A high value of RD expresses the proximity of *O* distribution versus the *R* or *M* distribution. 

### 3.1. An Exemplary Protein: Small-Conductance Mechanosensitive Channel—MscS (PDB ID 2VV5) 

The small-conductance mechanosensitive channel protein—MscS (PDB ID 2VV5) is the subject of a thorough analysis focused on the application of the FOD-M model to describe the structure of the membrane ion channel as an example of “reverse” hydrophobicity distribution versus micelle-like degradation.

**Figure 4:**
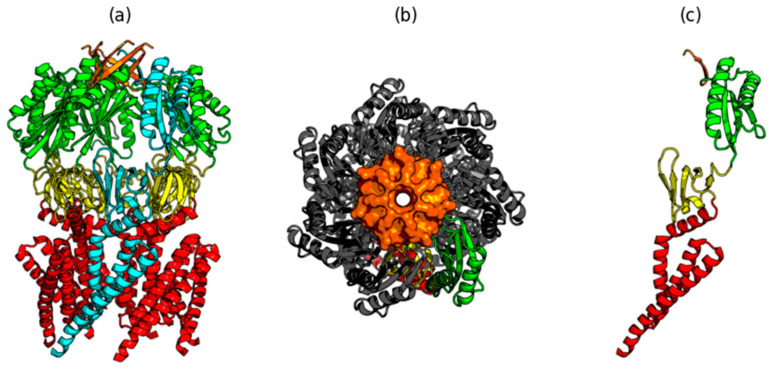
3D structure od MscS (PDB ID 2VV5). (**a**) red—dom1 (25–128), yellow—dom2 (129–181), green—dom3 (182–270), orange—dom4—beta-barrel (271–278), chain A distinguished as blue, (**b**) other orientations of the complex, gray—chains B–E, chain A—green, (**c**)—single chain—colors as in (**a**).

In a single chain, three domains are distinguished: domain 1 (27–128), domain 2 (129–178), and domain 3 (179–273) (from PDBSum [34]). The structure of the chain in question also includes a beta-structural fragment (27-1-279). These C-terminal fragments from all seven chains form the characteristic beta-barrel. The domain arrangement is visualized in Figure 4.

It should be noted that dom1 is a domain completely immersed in the membrane, while the others are exposed to the cytoplasmic environment.

The status of the domains, both in the complex and in a single chain, is presented by a set of RD values for the T-O-R relationship (the reference distribution for the O distribution is the T distribution and the R distribution) and for the T-O-M relationship (the reference distribution for the O distribution is the T distribution and the M distribution).

**Figure 5:**
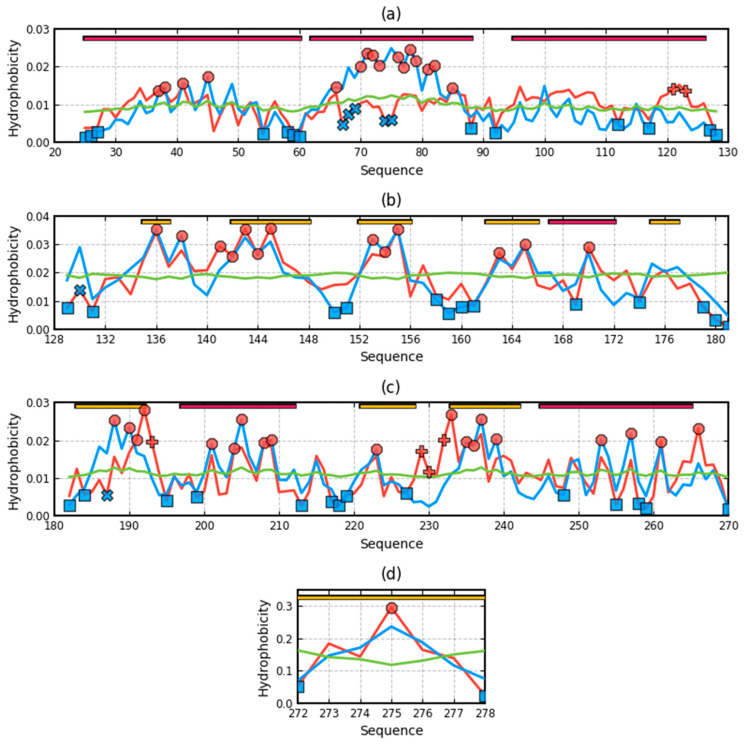
Profiles T, O, and M in domains of chain A treated as individual structural units (3D Gauss generated for each domain individually) (**a**)—dom1, (**b**)—dom2, (**c**)—dom3, (**d**)—beta-barrel. Distribution T—blue, O—red, M—green. Bars on top—presence of secondary forms: red—helix, yellow—beta-structure, red circles—residues potential hydrophobic core, blue X—residues of hydrophobicity deficiency—here residues in a channel, red “+”—residues of hydrophobicity excess on the surface potentially engaged in protein-protein complexation. The blue squares—residues on the surface representing low hydrophobicity—the status accordant with FOD criteria.

**Figure 6:**
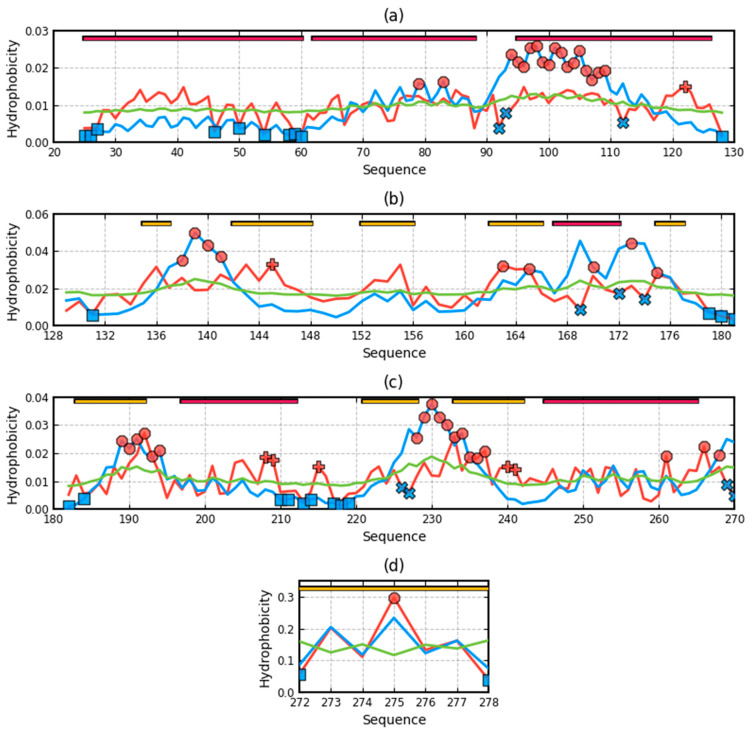
Profiles T, O, and M for chain A treated as part of the complex (3D Gauss function generated for the complete complex) (**a**)—dom1, (**b**)—dom2, (**c**)—dom3, (**d**)—beta-barrel Blue—T distribution, red—O distribution, green—M distribution. Bars on top represent fragments of secondary structure: red—helix, yellow—beta-structure. Red circles—residues potentially creating a hydrophobic core, blue “X”—residues of hydrophobicity deficiency—here residues engaged in channel building, red “+”—residues expressing hydrophobicity excess on the surface potentially engaged in protein-protein complexation. The blue squares—residues on the surface representing low hydrophobicity—a status accordant with FOD criteria.

The summary of the T, O, and M distribution in the chains constituting a part of the structural unit, which is the system of seven corresponding chain fragments included in a given domain, is shown in Figure 5. The graphic interpretation of the obtained results is presented in Figure 6.

It should be noted that the domain is treated in the form it takes for a given domain and the 3DG function is constructed only for a given domain. Here, the notation for the identification of an individual chain and “C” denotes the status of domains as part of complex is entered.

The status of the domains in the complex means that the 3DG function has spanned over all domains derived from the seven chains within the range of residues as given above.

When analyzing individual domains, it should be noted that dom1 is treated as a set of seven N-terminal domains distinguished in a single chain. The bold values in Table 1 indicate the optimal status for a given unit. They represent values closest to the reference distribution (T, R, or M). This situation is presented graphically in Figure 6. 

The status of the complex is expressed by a higher value of RD for the T-O-M relation. It suggests adaptation of hydrophobicity distribution according to the hydrophobic environment. However, the status of the individual chain represents the highest proximity versus the R distribution.

The status of the domains is differentiated. Dom1, dom2, and dom3 represent the status of highest proximity versus the M distribution. The highest one is observed in dom1. In dom1 two factors influence the status: exposure to the hydrophobic environment and the presence of a polar channel in the central part. The lowest RD values for dom2 and dom3 are the effect of only one factor influencing the hydrophobicity distribution, which is the presence of a channel. Analog values of RD for individual domains in a single chain represent the opposite situation. Dom2 and dom3 represent the status of a micelle-like form even for T-O-R, as well as for T-O-M relations. It means that the structure of these domains was reached according to a mechanism acting in the water environment. Dom1 is strongly different. Its structure seems to be difficult to be reached in the water environment, representing a highly improper status for a polar environment. Dom4—Beta-barrel—generated by the C-terminal fragments of seven chains represents the status accordant to T distribution—it means the micelle-like construction is present. 

### 3.2. Single Chain Analysis 

Dom2 and dom3 treated as individual structural units in a single chain (the 3DG function spanned over each of the domains separately) take the optimal form for micelle-like distribution, indicating values below 0.5 (the proximity of the O distribution to the T distribution), regardless of the adopted model. The values of RD for the T-O-M relationship also indicate the proximity of the O distribution to the T distribution.

Domain 1 in the form of an individual structural unit indicates an arrangement indicating the absence of a hydrophobic core. The R distribution is the uniform distribution of hydrophobicity throughout the domain. In this domain form, the effect of a factor of the “reverse” distribution type is not visible. Domain 2 individually, despite the environmental correction, shows significant adaptation to the T distribution. 

Dom2 and dom3 are treated as individual structural units (3D Gauss function generated for each domain individually) despite the environmental correction (M component present) representing the status close to T distribution. 

It can, therefore, be speculated that the self-folding chain shapes domains 2 and 3 as independent structural units with a hydrophobic pattern consistent with the 3D Gauss distribution. On the other hand, dom1 in the water environment does not form a micelle-like system, showing as an individual domain and configuration close to FOD-M.

A visual analysis of the T, O, and M profiles for dom2 and dom3 domains with a highly aligned order with the FOD model shows a negligible representation of the M distribution for the description of the distributions in these domains. The M distribution for domain 1 (Figure 6) shows a significant correction of the results.

### 3.3. Analysis of Complex 

High values of RD indicate a large distance of the O distribution to the T distribution. This means proximity to the second reference distribution. Assessment of the status of individual domains treated in the quaternary version (3D Gauss function generated for a set of chain fragments corresponding to domains in a seven-chain system) of the complex shows the lowest distribution distance of O to the M distribution. This state is visualized by the sets of T, O, and M profiles for the domains in question in their quaternary form (Figure 6). 

The most representative of the O distribution is the representation of the M distribution in domain 1. In the remaining domains, the M distribution does not make any corrections. This is due to the relatively high ordering of the O distribution in relation to the T distribution. It is most visible in the case of the beta-barrel domain, where the compatibility of T and O is very high. The M distribution does not bring anything valuable, but it does not disturb the interpretation resulting from the analysis in the T-O-R system (Table 1).

**Figure 7:**
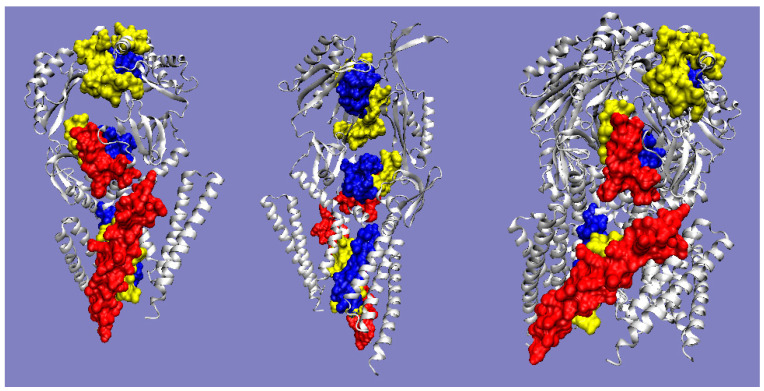
3D presentation of the complex. Chain A distinguished in colors: red—hydrophobicity excess, blue—hydrophobicity deficiency, yellow—status accordant with the model. **Left**—surface view, **central**—channel view, **right**—complex. The white chains—other chains.

The presence of chain fragments of O status accordant with T distribution should be noted. These fragments are as follows: dom1: 70–90, dom2: 157–165, dom3: 195–202. This supports the accepted consensus status for both discussed environments. Figure 1 distinguishes this area as grey. The discussed chain fragments in 3D presentations are given in Figure 7. 

Figure 7 visualizes fragments of the status identified according to profiles shown in Figure 6. Fragments distinguished (Figure 6) characterized by hydrophobicity excess—red: 25–60, 120–125 in dom1 and 143–156 in dom2. Blue—fragments identified as hydrophobicity deficiency (in respect to T distribution): 90–110 in dom1, 136–142, 166–175, in dom2 and 188–194, 225–237 in dom3. Fragments distinguished as yellow—fragments of hydrophobicity level accordant with both models.

### 3.4. Other Exemplary Membrane Proteins as Membrane Channels 

To complete and extend the list of examples, three membrane proteins acting as channels were also analyzed: mechanosensitive channel protein—MscS (PDB ID 4Y7K), helicobacter pylori MscS (closed state)—MscS (PDB ID 4HW9), and rhodopsin (PDB ID 1U19). The selected proteins differ in composition—the number of chains included in the complex and the type of transporting molecules, as well as the presence of a domain directed towards the cytoplasm. This domain is not present for 1U19. The discussion of these proteins is limited to giving the RD values for the T-O-R and T-O-M relationships designated for the domains. The results are summarized in Table 2.

**Figure 8:**
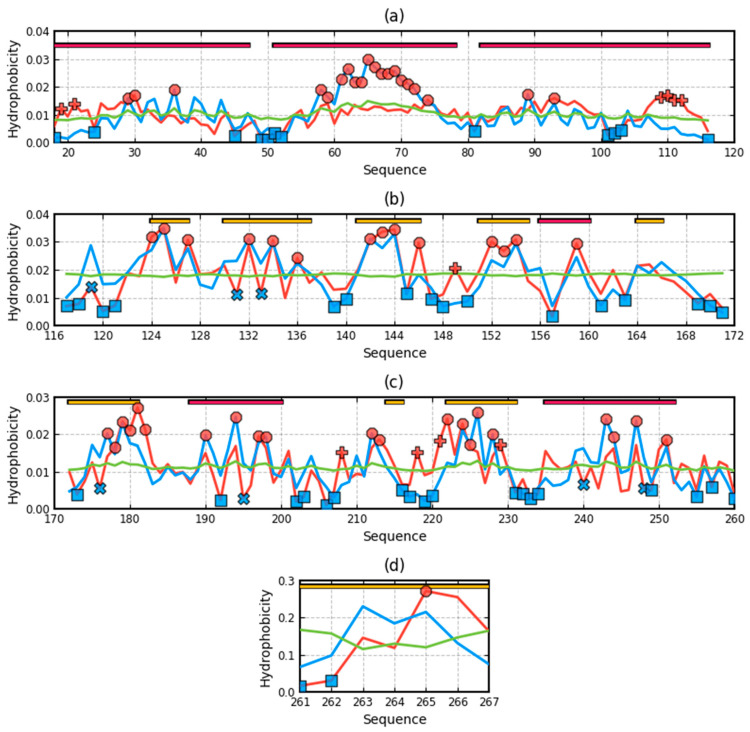
Profiles T, O, and M in Romains of 4HW9-3D Gauss function generated for each domain individually (**a**)—dom1, (**b**)—dom2, (**c**)—dom3, (**d**)—dom4—beta-barrel. T distribution—blue, O distribution—red, M distribution—green. Top bars: yellow—beta-structure, yellow—helix. Red circles—residues potentially engaged in hydrophobic core construction, blue “X”—residues of hydrophobicity deficiency—here residues engaged in channel construction, red “+”—hydrophobicity excess potentially engaged in protein-protein complexation—here—the interaction with membrane. The blue squares—residues on the surface representing low hydrophobicity—a status accordant with FOD criteria.

**Figure 9:**
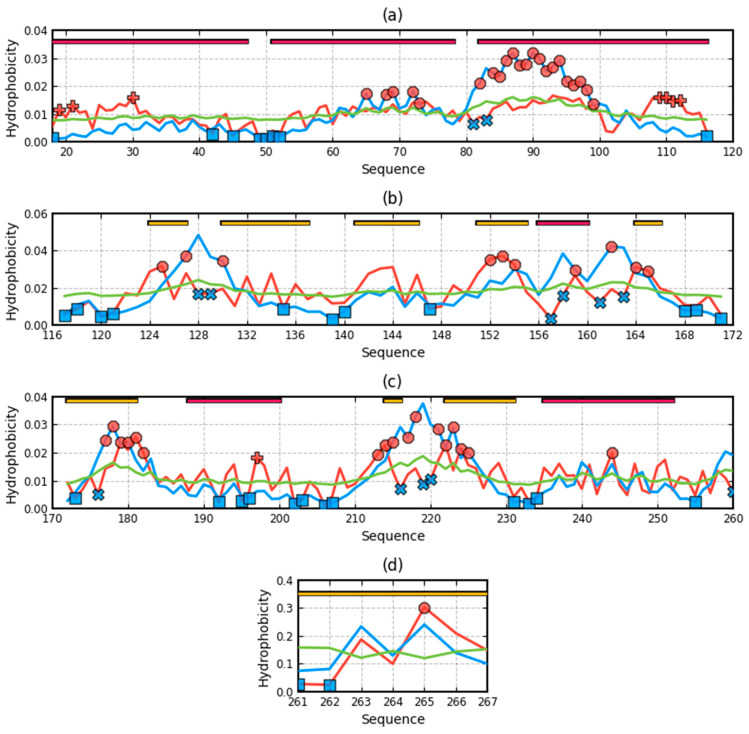
Profiles T, O, and M in 4HW9-3D Gauss function generated for the complex. (**a**)—dom1, (**b**)—dom2, (**c**)—dom3, (**d**)—dom4—beta-barrel. T distribution—blue, O distribution—red, M distribution—green. Top bars: yellow—beta-structure, yellow—helix. Red circles—residues potentially engaged in hydrophobic core construction, blue “X”—residues of hydrophobicity deficiency—here residues engaged in channel construction, red “+”—hydrophobicity excess potentially engaged in protein–protein complexation—here—the interaction with membrane. The blue squares—residues on the surface representing low hydrophobicity—a status accordant with FOD criteria.

**Figure 10:**
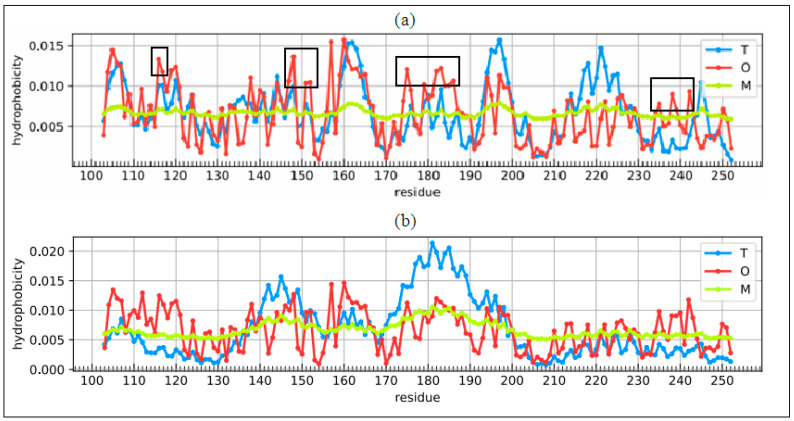
Characteristics of dom2 domain—(**a**)—profiles T, O, and M identified in dom2. The residues engaged in the P–P interaction are designated. (**b**)—profiles T, O, and M in domain dom2 after complex formation.

**Figure 11:**
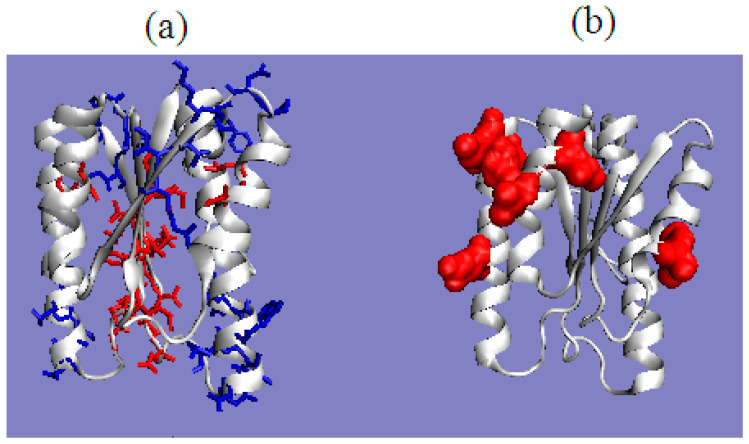
3D visualization: (**a**)—dom2 with the highlighted zones: hydrophobic core—red, surface—blue, (**b**)—a structure of dom2 with the highlighted residues (red) representing a local hydrophobicity excess—in a complex engaged in P-P interaction.

Table 2. lists RD parameters determined for the T-O-R and T-O-M relations in membrane and cytoplasmic domains. Appropriate profiles are shown in Figure 8, Figure 9, Figure 10 and Figure 11. Summary of RD values determined for the membrane and cytoplasmic domains of proteins serving as channels for the transport of molecules/ions through the membrane. The results for the membrane domains indicate a significant share of the hydrophobic environment in shaping the hydrophobicity distribution consistent with the FOD-M model. The high incompatibility of these distributions for the T-O-R relationship results from the presence of two factors disturbing the system expressed by the 3DG function. These two factors are the exposure of hydrophobic residues and a much lower than the expected presence of a hydrophobic system in the center of the domain where the channel is present, and thus low levels of hydrophobicity.

The lower difference between the status expressed for the domain exposed to the aquatic environment is due to the fact that only one factor in the form of decreased hydrophobicity in the central part of the domain (channel) favors a different decomposition than 3DG. The surface in the domain exposed to the cytoplasm shows surface coverage with polar systems. 

Figure 8 and Figure 9 reveal the decreasing of differences between RD for T-O-R and T-O-M going from dom1 to dom3 and even to dom4 (Beta-barrel). The profiles on Figure 8a and Figure 9a—particularly, profile M, follow the changes of hydrophobicity level—O. However, only in the presence of channel causes, the discrepancy gets smaller. The dom4 (beta-barrel) entirely exposed to a water environment with a small diameter in the channel represents even the status micelle-like with RD < 0.5. 

### 3.5. Speculation on the Process of Chain Folding for the Proteins Acting in Hydrophobic Environment 

Using the example of a protein belonging to the mechanosensitive channel group—the MscS (PDB ID 4Y7K), a scenario for the formation of the structure present in the complex can be proposed. Discussing the possible folding scenario of the protein in question, attention should be focused on the status of dom2 when it is regarded as an individual structural unit.

Dom2 shows the RD values below 0.5 for both the T-O-R and T-O-M relations (Table 3). This means that there is a micelle-like arrangement in this domain. Such a domain spontaneously folds in the presence of a polar aquatic environment. Nevertheless, on the profiles T and O, it is possible to identify positions that show locally an excess of hydrophobicity. The residues with this status—as follows from the adopted distance criterion—interact with another chain due to the hydrophobic interactions (Figure 10). Eliminating the residues involved in the P-P interaction reduces the RD (T-O-R) value to 0.418. The status of the residues involved in the P-P interaction is expressed by the value of RD = 0.641. According to the fuzzy oil drop model, such an arrangement makes it possible to prepare a protein (domain) to complex another chain (protein)—residues of the status of local excess of hydrophobicity on the surface (Figure 10a).

The comparison of the profiles (Figure 10) indicates a radical change in the status of dom2 in the complex, despite the exact same structure in both cases. According to the fitted 3D Gaussian function, the individual chain sections and residues are evaluated differently, changing the status of the domain dom2. The segment 170–190 in the individual domain shows a local excess of hydrophobicity, which in the complex turns out to be too low to generate the expected hydrophobic core, thus creating a channel surface. Simultaneously, the status of these residues in an individual domain makes them ready for the complex in the other domain. The example given is the C-terminal fragment that exhibits an excess level of hydrophobicity used for chain-chain interactions. In the case of a complex, the local excess recorded at positions around 150 in the individual domain is a part of the local maximum in the complex.

**Figure 12:**
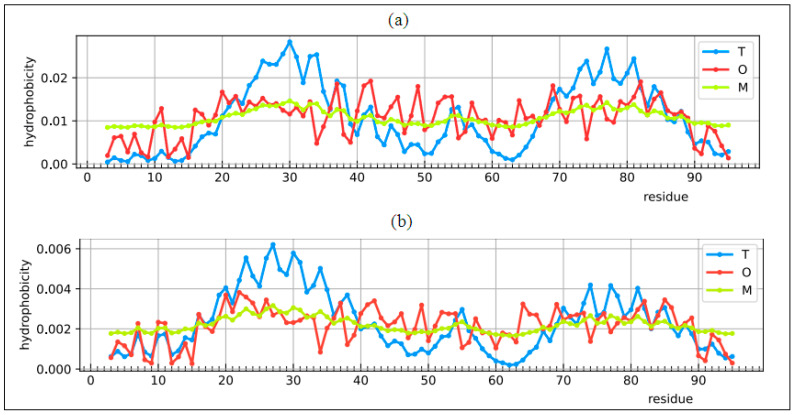
Characteristics of domain dom1 (**a**)—profiles T, O, and M for dom1 regarded as an individual structural unit (3D Gauss function fitted for the individual domain dom1). (**b**)—profiles T, O, and M for the domain dom1 regarded as an element of a complex.

The domain dom1 (3–95), composed mainly of helices, regarded as an individual structural unit shows RD = 0.706 for the T-O-R and RD = 0.754 for the T-O-M relations, respectively. Such a high value of RD suggests the presence of significant incompatibilities between O and T distributions that cover the entire domain (Figure 12) This discrepancy is a result of both a hydrophobicity deficiency in the sections that should be a part of the hydrophobic core, as well as an excess in the sections where a low level of hydrophobicity is expected—i.e., on the surface. This means that neither the core was generated nor the polar surface. According to the fuzzy oil drop model, the folding of this domain as an individual structural unit requires the presence of a factor that excludes obtaining a globular structure with a centric core. A high value of RD for the T-O-M relation suggests the presence of an external field directing the folding process to the exposure of hydrophobic residues on the surface. The presence of an environment other than a polar external environment—the hydrophobic one—is expected in the case of this domain. 

The profile analysis (Figure 12) suggests a similar deficiency of hydrophobicity in the position of the expected centric hydrophobic core; however, in the complex, the status of the 60–100 helix occupying a position on the surface of the complex represents a compliance status with the expected one. On the other hand, the N-terminal helix, which occupies a position closer to the center of the complex, shows a significant deficit of hydrophobicity due to the presence of a channel. The differences between the profiles in Figure 12a,b result from the relative orientation of the helices towards the center of the object. High values of RD for dom1 regarded as an individual structural unit indicate that the orientation comes from the surrounding environment already during the formation of this structure. The high consistency of the distribution in the T-O-M relation suggests the participation of the hydrophobic environment in folding this domain. The exceptional status of fragment 95–102, which can be considered unstructured, may result from the impossibility of incorporating this section into dom2 (it is an ordered system—incorporation of this chain fragment is disfavorable). This section probably takes this form in the final stage of the construction of the complex (dom1 already anchored in the membrane), acting as a hinge enabling the potentially limited mobility of dom2 (cytoplasmic environment) against the stiffened, much more membranous dom1. The importance of the N-terminal fragment is specific: there is a discrepancy in an individual domain, which turns out to be a match in the complex structure. The given scenario is hypothetical. However, it is a result of the rules and principles of the characteristics of the environment in which a given domain (complex) is probably created. 

### 3.6. Proton Pump (PDB ID-1U19)

**Figure 13:**
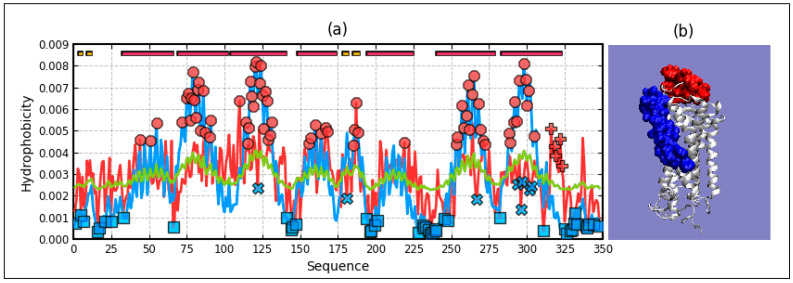
Characteristics of 1U19 (**a**)—profiles T, O, and M in domains in 1U19-3D Gauss function generated for chain A Top bars: red—helix. Red circles—residues potentially engaged in hydrophobic core construction, blue “X”—residues of hydrophobicity deficiency—here residues engaged in channel construction, red “+”—hydrophobicity excess potentially engaged in protein-protein complexation—here—the interaction with membrane. The blue squares—residues on the surface representing low hydrophobicity—status accordant with FOD criteria. (**b**)—3D presentation with residues distinguished—hydrophobicity deficiency (“X” on profiles)—blue, hydrophobicity excess (“+” on the profiles)—red. Colors are shown only for chain A.

The analysis of the T, O, and M profiles shows the consistency in the so-called “gray” zone, where the distributions of T, O, and M agree with each other (comparison with Figure 1). This zone applies to a local maximum of 100–130 or 250–280 in a proton pump (Figure 13). 

On the other hand, there is a clear reduction in the level of hydrophobicity in the central location (expected maximum hydrophobicity) and a segmented excess of hydrophobicity in the areas in contact with the membrane. 

### 3.7. Potassium Channel Subunit Alpha Kvlqt1 (3HFE) as an Example of a Channel-Like Domain

A trimeric form of the kv7.1 a domain tail voltage-gated potassium channel subunit kv7.1 producing slow voltage-gated potassium channel subunit alpha kvlqt1, kqt-like 1—as described by crystallographers [27] is the most substantial part of the ion channel discussed in this paper. 

The question is to what extent the predisposition to construct this form of biological activity is already encoded in the amino acid sequence of the 27 aa helix. The analysis of the T and O profiles indicates a uniform hydrophobicity distribution, where a high level of hydrophobicity is observed in the area of the expected low levels of hydrophobicity. Likewise, in the center of the expected hydrophobicity concentration, a significantly lower level is present. The way in which such a constructed helix enters the trimer system gives a new character that can be traced by analyzing the status of the helices in the complex.

**Figure 14:**
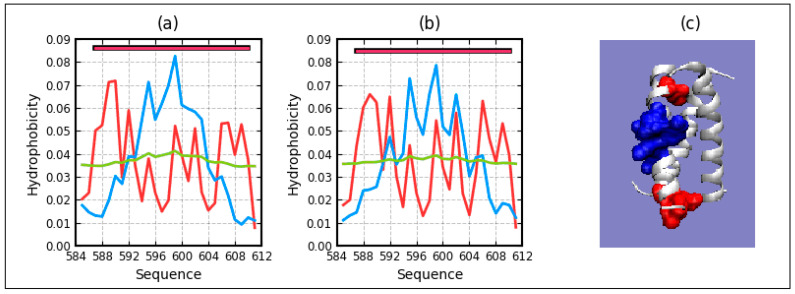
Potassium channel subunit alpha kvlqt1 Profile T (blue), O (red), M (green) (**a**) chain A as an individual structural unit, (**b**) chain A as part of complex, (**c**) 3D presentation of the complex with residues of hydrophobicity deficiency—red, of hydrophobicity excess—red. Colors are only shown for chain A.

This status changes in the form of magnifying the specificity of a single chain distribution. Quantitatively, the monomer and trimer structures are characterized by the following parameters: individually treated chain RD T-O-R = 0.763 and T-O-M = 0.747. The single-chain status indicates the proximity of the distribution to the uniform distribution. On the other hand, trimer status is expressed by the following characteristics:

RD for T-O-R = 0.668 and for T-O-M = 0.708. By comparing the results, there is a tendency towards a structure showing the presence of a channel-like form which, for obvious reasons, cannot be identified in a single chain. Graphically, the distributions of T, O, and M are given in Figure 14. 

Speculation on the mechanism of structure generation of this system is quite difficult. Analysis of the amino acid sequence on the basis of the secondary structure preferences points out the beta-structure as the dominating one. The analysis of chameleon sequences gives the predisposition for helix = 1.28, for beta-structure = 0.956 and for RC = 0.628 respectively [34]. 

The analysis of this structure based on the fuzzy oil drop model suggests the folding process as it could happen in a vacuum without any influence of the environment pointing out the R reference distribution as the closest one versus the distribution O. 

### 3.8. Test Molecules 

The analysis of protein structures representing different biological functions showing high compatibility of the T and O distribution—and thus representing the micelle-like structure, carried out with the FOD-M model in mind, is aimed at verifying the degree of universality of the introduced modification. It is expected that the correction for the effect of the membrane environment should not affect the results on the water-soluble and solubilizing proteins. Proteins operating in the aqueous environment were selected, although one of them exhibited an RD > 0.5 (0.5 was taken as the cut-off value). These exemplary proteins are the titin domain (PDB ID 1TIT) and lysozyme (PDB ID 1LZ1) as shown in Table 3.

**Figure 15:**
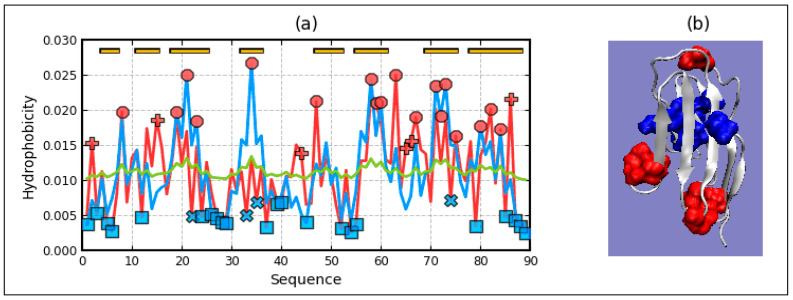
Hydrophobicity distribution in titin. (**a**) T (blue), O (red), and M (green) as appear in titin. The blue squares—residues on the surface representing low hydrophobicity—status accordant with FOD criteria. (**b**) 3D structure of titin with residues of local hydrophobicity deficiency (“X” on the profiles in (**a**)) and local hydrophobicity excess (“+” on the profiles in (**a**))—red.

The titin status described by the FOD model and the FOD-M model is expressed with the RD values for T-O-R: 0.424 and for T-O-M: 0.477. Both of these values indicate the presence of a spherical micelle-like structure with a centrally located core and a polar shell. The introduction of modification of the external field increases slightly the value of RD, but the status of the protein still remains unchanged despite this correction. One shall keep in mind that the residues distinguished in Figure 15, though representing local deficiency/excess the classification of titin, do not change the global status of the domain as representing the hydrophobicity status accordant to micelle-like construction (RD < 0.5). It means the discrepancy observed here is not significant. The status of titin is classified as representing the presence of a centric hydrophobic core. 

The lysozyme status described by RD for the T-O-R relationship is 0.529. It shows a slight local incompatibility of the distribution with respect to the micelle-like distribution. Eliminating the catalytic residues (E35 and D53) and the C128 residue from the calculation changes the RD value to the level of 0.493. This shows that the presence of catalytic residues imposes a local disturbance in the order of the O distribution in relation to the T distribution. Position C128, which is part of a disulfide bond located very close to the surface, introduces a local excess of hydrophobicity. The elimination of the E35, D53, and C128 residues identifies the part of the protein that meets the idealized degradation conditions that guarantee the solubility of the protein, and at the same time suggests that the protein was folded according to the 3DG force field.

The application of the FOD-M formalism for lysozyme shows the RD value for the complete protein of 0.598, while for the protein lacking the mentioned residues the value is 0.433. The interpretation of the status of this enzyme, devoid of the mentioned residues, indicates a complete micelle-like arrangement of hydrophobicity. On the other hand, the introduction of the environmental modification indicates that the O distribution is close to the M distribution. Is there any channel within the lysozyme? No, but a cavity complexing a substrate (peptidoglycan) with an elongated structure, where the interaction occurs along the entire length of the substrate molecule (especially at its end fragments) only requires an elongated cavity.

**Figure 16:**
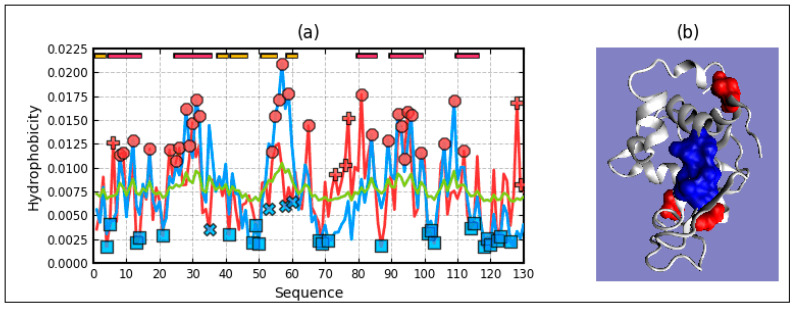
Profiles in human lysozyme. (**a**) T—blue, O—red, and M—green profiles. The blue squares—residues on the surface representing low hydrophobicity—status accordant with FOD criteria. (**b**)—3D structure of lysozyme. The residues with hydrophobicity excess ((“+” on the profiles in (**a**))—red, residues with local hydrophobicity deficiency (“X” on the profiles in (**a**))—blue.

The local maladjustment of the O distribution to the T distribution was interpreted as the local inability to generate a micelle-like distribution. The presence of a ligand during the folding process of the polypeptide chain, the presence of which in the final version of the folded protein often determines its biological activity, has also been proposed [35]. It was speculated that the ligand (substrate) showing its characteristic level of hydrophobicity in the external field as expressed by the 3D Gauss function competes with the folding chain to occupy the appropriate location. Such a model helps to explain the presence of high specificity for this type of interaction (complexation). In the light of the results concerning the analysis of the lysozyme structure, it can be assumed that there is a factor (not necessarily a ligand or a substrate) which, by changing the properties of the surrounding water, allows for a different ordering of the polar–nonpolar group relations, which favor the formation of a local order consistent with the modified external field, by changing the properties of the surrounding water. A visual assessment of the T, O, and M distributions suggests that the local maximum 51–63 (beta-structure segments included in the only beta-sheet in this protein) has a specificity, which is taken into account by the FOD-M model, representing a much lower level of hydrophobicity (two catalytic residues are present in this fragment E35 and D53) (Figure 16—particularly Figure 16b—the blue fragment with catalytic residues). 

The analysis of this phenomenon requires further research on a larger group of proteins. The proteins with local discordances between O and T distributions that appear to be related to biological activity seem to represent the specific coding system [35]. 

## 4. Discussion 

The issue of the presence of channels is the subject of many analyzes. A model of a rolling ball with a variable radius to identify the channel in the protein structure was used in a program called HOLE [36]. The presence of the tunnels was identified on a large scale [37]. These methods allow for the identification of tunnels, although they do not allow for quantitative analysis, including comparative analysis. The modification of the FOD model to the FOD-M form, taking into account the presence of the membrane environment treated as “inverse” to the influence of the aqueous environment, allows not only quantitative comparative analysis of individual proteins, but also enables speculation about the mechanisms accompanying the process of folding their preferences actively influence the final shape and function. The basic assumption of the fuzzy oil drop model is the active participation of the aquatic environment in shaping the protein structure. The presence of the membrane as the environment for the activity of membrane proteins has an obvious influence on the structure and activity of membrane proteins. Other structural forms of these proteins are currently being analyzed in order to identify differences in the world of membrane proteins and their specificity, both functional and structural.

The speculation concerning the folding process takes into account the active participation of the external force field in all versions of the FOD model. Depending on the form of the external force field, different final models can be obtained. Protein folding directed by a certain form of external force field may lead to a defined aim-oriented structural form with specificity coded in its structure. The analysis of amyloid structures reveals the planar (not 3-dimensional) structure of individual chains present in fibrils. It is assumed that such structures can be obtained in an external force field of the 2D Gauss function directing the hydrophobic residues towards the central part; however, in a planar form [22]. The specificity of the environment changing this form of water activity is currently under consideration. Similarly, the folding of proteins, which is specifically dedicated to acting in a membrane environment that can be reached in a folding simulation using the FOD-M force field proposed in this paper. The multi-criteria optimization has been proposed in [38] to take into account the internal force field (non-bonding interaction) optimization and influence of the external force field (of different characteristics) to introduce the possible synergy in the folding process. Probably some other forms of external influence are possible to simulate the folding process. The introduced model makes it possible to ask the question: why do the proteins fold the way they do? The evaluation of the participation of the external force field can be used to identify causes of protein misfolding in silico. The parameterization used in most widely used force fields to predict protein structures is based on the aquatic environment. Hence, taking into account a non-polar environment can help identify the cause of a protein misfolding [39].

## 5. Materials and Methods 

### 5.1. Data 

The subjects of the analysis are the proteins listed in Table 4, where their short characteristics are given. 

The analysis of three membrane proteins from the MscS group (Small Conductance Mechanosensitive Ion Channel) provided the basis for a modification in the fuzzy oil drop model, also extending its application to membrane proteins. The analysis also included a non-channel transmembrane protein. The protein acting as a proton pump in the central part of the molecule binds retinol—a fairly large molecule. The cavity that binds this molecule is of considerable size, creating free space in the central part. Therefore, a modified version of the FOD-M model was also used to describe this molecule, where M stands for membrane.

The helix bundle is a distinctive structural element often found in a membrane-anchored domain. Therefore, the structures of the three-helix bundles were included in the analysis of the structure of membrane proteins. The presence of titin is dictated by the need to verify the effect of the introduced modification on the protein, which does not require the correction to be introduced. Titin shows a high agreement of the observed hydrophobicity distribution (O) with the expected hydrophobicity distribution (T).

Lysozyme is an example of a water-soluble protein with local incompatibility of the distribution of T (idealized one) and O (observed distribution). The use of FOD-M for the description of these two proteins is aimed at verifying the universality of the introduced extension, including the presence of a highly hydrophobic cell membrane environment. The introduction of the discussed modification does not change the status of proteins with distributions highly consistent with the distribution of the fuzzy oil drop model.

### 5.2. Programs Used

The graphics were produced using the program PyMol [40,41] and VMD [42]. The server for the generation of hydrophobicity profiles is available at [43]. 

## 6. Conclusions

In the present work, the modifications to the fuzzy oil drop model have been positively verified by introducing a supplement that takes into account the presence of a non-polar environment. The correction in the form of (T_Max_ − T_i_)_n_ turned out to express the presence of a hydrophobic external field. It is assumed that the introduction of the K coefficient, the variability of which can be applied to various non-membrane external conditions, can express this variability of conditions. Thus, it becomes a representation of the contact environment-protein surface for the changing nature of the environment. The general notation taking into account the variability of the polarity/non-polarity characteristic (M-distribution—from the word Membrane) of the environment is expressed as follows:

Mi = [Ti + K(Tmax − Ti)_n_ ]_n_ (6)

and in more general form:

M = [3DG + K × (MAX_3DG_ − 3DG)_n_]_n_ (7)

The notation used uses the abbreviation 3DG expressing the 3D Gauss function. MAX_3DG_ means the maximum value in the determined idealized distribution (also called T). The index n denotes the normalization of the distribution. The bold part of the phrase expresses the influence of the environment. The role of the K parameter is to express the degree and strength of the impact of the variable characteristics of the environment. The value of K = 0 means the influence solely of the polar water environment. The given expression for K = 1 applies to the membrane protein system acting in the ion channel. The deviation from the decomposition of 3DG comes from the need to expose hydrophobic residues to the surface (contact with the hydrophobic environment of the membrane) and from the concentration of polar residues in the central part of the membrane protein complex. This arrangement is the inverse of the 3DG (T) distribution used to describe the hydrophobic nucleus status of water-soluble globular proteins.

The study also showed no negative impact on the assessment of the status of proteins with a high conformance distribution between the T and O distribution. Even the K = 1 value in the case of proteins with a high degree of order conforming to the 3DG distribution does not adversely affect their evaluation.

The currently developed modification extending the application of the fuzzy oil drop model to membrane proteins is the second modification of this model. A previously introduced modification applied to the amyloid structures is a form of 2DG. This notation reflects the distribution of hydrophobicity within a single chain—the component of the amyloid fibril, where all the chains represent a flat structure. This model can be referred to as FOD-A, where A is amyloid [22].

## Figures and Tables

**Table 1:** RD values for the T-O-R and T-O-M relations for the distinguished domains in the Small-conductance mechanosensitive channel—MscS (PDB ID 2VV5). Values given as bold—optimal status of a given unit. DOM4 is the special one—it is created by a C-terminal fragment of all the chains present in the complex forming the common β-barrel. For this reason, the cells in the right columns presenting the individual chains are empty.

	Complex		Individual Chain	
	T-O-R	T-O-M	T-O-R	T-O-M
Complete Unit	0.759	**0.764**	**0.763**	0.747
DOM 1 25–128	0.746	**0.776**	0.739	**0.751**
DOM 2 129–181	0.552	**0.740**	**0.383**	**0.372**
DOM 3 182–270	0.652	**0.683**	**0.402**	**0.446**
DOM 4 271–278	**0.146**	0.164		

**Table 2:** *RD* values for discussed proteins. The relation T-O-R and T-O-M are presented for complex and individual domains.

PDB -ID	Membrane Domain			Cytoplasmic Domains		
	Fragment	T-O-R	T-O-M	Fragment	T-O-R	T-O-M
4HW9	18–116				0.847	0.821
Complex		0.782	0.775	117–171	0.625	0.65
Chain A					0.339	0.326
Complex		0.787	0.813	172–260	0.627	0.682
Chain A		0.752	0.768		0.472	0.503
Complex				261–	0.154	0.209
Chain A					0.208	
1U19-dimer	complex	0.718	0.747			
Chain A		0.679	0.738			
Chain B		0.676	0.73			

**Table 3:** Characteristics of the mechanosensitive channel—MscS (PDB ID-4Y7K) by the values of parameter RD on the specified domains for T-O-R and T-O-M relations. Top line designates a status of the complex composed of 5 chains regarded as a structural unit. Complex {A–E}—status of domain build by five domains representing each chain. Chain A in complex—the status of the domain regarded as a part of a complex composed of 5 domains, Individ chain A –status of the domain specified as a separate one in the individual chain.

PDB-ID	Membrane Domain			Cytoplasmic Domains		
	Fragment	T-O-R	T-O-M	Fragment	T-O-R	T-O-M
4Y7K						
Complex {A–E}	3–95	0.678	0.717		0.662	0.687
Chain A in complex				103–252		
		0.645	0.695		0.653	0.682
Indiv. chain A		0.706	0.754		0.461	0.494

**Table 4:** The set of proteins discussed in the present work. A brief description and reasons for presence in the presented analysis are given.

PDB-ID	Protein	Characteristics	Structural Specificity	Ref
2VV5	Small-conductance mechanosensitive channel—MscS	Open MscS-7 chains	Model for FOD modification-7 chains-channel	[23]
4Y7K	mechanosensitive channel—MscS	Closed MscS-7 chains	Model for FOD modification-7 chains-channel	[24]
4HW9	Helicobacter pylori mscs (closed state)—MscS	Closed MscS-5 chains	Model for FOD verification-5 chains-channel	[25]
1U19	RhodopsinR	Proton pump	Transmembrane proteins	[26]
3HFE	Trimeric kv7.1a domain	Helix coiled coil	coiled-coil	[27]
1TIT	Titin	Single domain	High accordance with FOD	[28]
1LZ1	Lysozyme	One chain enzyme	Local discordance with FOD	[29]

## Data Availability

Data can be available on request addressed to Correspondence Author.

## Abbreviations

FOD	Fuzzy oil drop model
FOD-M	Modified fuzzy oil drop model—the membrane environment included
D_KL_	Kullback-Leibler divergence entropy
RD	Relative Distance
PDB	Protein Data Bank
T	Theoretical hydrophobicity distribution—according to 3D Gauss function
O	Observed hydrophobicity distribution—calculated according to Levitt’s definition
R	Unified hydrophobicity distribution—all residues represent the same level of hydrophobicity
M	Distribution based on the presence of membrane environment
T-O-R	RD relation for O distribution with T and R distributions treated as reference distributions
T-O-M	RD relation for O distribution with T and M distributions treated as referenced distributions
FOD-A	Fuzzy oil drop model modified for amyloid description
3DG	3 dimensional Gauss function
2DG	2 dimensional Gauss function
Dom#	Domain with the number according to the position the chain
